# Synthesis of a novel isotopically labelled standard for quantification of γ-nonalactone in New Zealand Pinot noir via SIDA-SPE-GC–MS

**DOI:** 10.1007/s00216-023-04789-2

**Published:** 2023-06-13

**Authors:** Gillean C. Miller, David Barker, Lisa I. Pilkington, Rebecca C. Deed

**Affiliations:** 1 School of Chemical Sciences, University of Auckland, Auckland, 1010 New Zealand; 2 The MacDiarmid Institute for Advanced Materials and Nanotechnology, Wellington, 6012 New Zealand; 3 Te Pūnaha Matatini, Auckland, 1142 New Zealand; 4 School of Biological Sciences, University of Auckland, Auckland, 1010 New Zealand

**Keywords:** Aroma, γ-Nonalactone, SIDA, Pinot noir, Wine

## Abstract

γ-Nonalactone is a linear aliphatic lactone ubiquitous in wine, associated with coconut, sweet, and stone fruit aroma descriptors. Little research has been conducted looking at the importance of this compound to New Zealand (NZ) wine aroma. ^2^H_2_^13^C_2_-γ-Nonalactone, a novel isotopologue of γ-nonalactone, was synthesised in this work for use in a stable isotope dilution assay (SIDA) for quantification of γ-nonalactone in NZ Pinot noir wines for the first time. Synthesis was carried out using heptaldehyde as the starting material, and ^13^C atoms and ^2^H atoms were introduced via Wittig olefination and deuterogenation steps, respectively. The suitability of this compound as an internal standard was demonstrated by spiking model wine at normal and elevated conditions during sample preparation, with subsequent analysis via mass spectrometry showing stability of ^2^H_2_^13^C_2_-γ-nonalactone. A model wine calibration, with concentrations of γ-nonalactone from 0 to 100 µg L^−1^, was shown to have excellent linearity (*R*^2^ > 0.99), reproducibility (0.72%), and repeatability (0.38%). Twelve NZ Pinot noir wines, representative of a range of NZ Pinot noir–producing regions, prices, and vintages, were analysed by solid-phase extraction–gas chromatography–mass spectrometry (SPE-GC–MS). The concentrations of γ-nonalactone ranged from 8.3 to 22.5 µg L^−1^, the latter of which was close to the odour detection threshold of this compound. These findings provide a basis for further research into γ-nonalactone and its impact on NZ Pinot noir aroma and provide a robust method for the quantification of this compound in Pinot noir.

**Graphical abstract:**

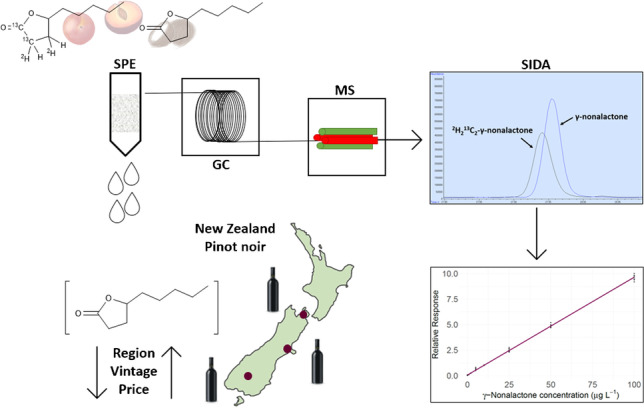

**Supplementary Information:**

The online version contains supplementary material available at 10.1007/s00216-023-04789-2.

## Introduction

**Fig. 1:**
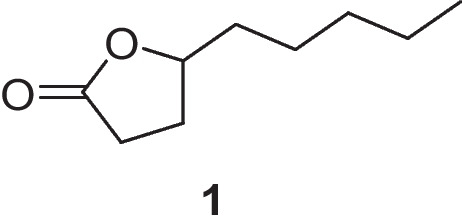
Chemical structure of γ-nonalactone **1**

The aroma profile of grape wines is largely determined by the impact of volatile aroma compounds, formed by the grape, during fermentation and processing, or through maturation and ageing. These aroma compounds contain a wide variety of functional groups and include acids, alcohols, esters, lactones, and thiols to name a few [1]. Lactones are common in wine and generally consist of a cyclic ester, formed by intramolecular cyclisation of a corresponding alcohol and carboxylic acid. Saturated linear aliphatic γ-lactones have historically been overlooked as wine aroma compounds [2]. Coconut, stone fruit, and sweet are among the descriptors attributed to these compounds in wine, and particularly high concentrations have been found in red wines and sweet white wines, such as noble rot wines with the influence of *Botrytis cinerea* [2-5]. γ-Nonalactone (Fig. 1) is arguably the most ubiquitous and significant of these lactones identified in wine, with an estimated odour detection threshold (ODT) of 30 µg L^−1^ for the racemic mixture [6]. First identified in wine in 1974 [7], γ-nonalactone has subsequently been found in a range of different wines, with concentrations as high as 971 µg L^−1^ in a sample of Verdejo wine [8]. γ-Nonalactone has also been quantified well above its ODT in wines made using grape dehydration techniques, including icewines and fortified wines (reported maxima of 179 and 539 µg L^−1^, respectively) [8, 9]. γ-Nonalactone has been shown to have synergistic interactions with other aroma compounds, including similar saturated linear aliphatic lactones γ-octalactone, γ-decalactone, γ-undecalactone, and γ-dodecalactone, thus potentially contributing to wine aroma even when present below its ODT [10].

Sensory analysis suggested that (*R*)- and (*S*)-enantiomers of γ-nonalactone have similar aroma descriptors, with distinct coconut notes [11]. The (*S*)-enantiomer had a significantly lower ODT in red wine (91 µg L^−1^) compared to the (*R*)-enantiomer (284 µg L^−1^) and is therefore thought to be a more powerful odorant. Cooke et al. [11] analysed a range of wines using chiral gas chromatography–mass spectrometry (GC–MS). These wines had a higher proportion of the (*R*)-enantiomer, with botrytised white wines showing the greatest proportion (75% (*R*)-enantiomer), on average [11]. Similar proportions were reported in Bordeaux Merlot and Cabernet Sauvignon wines (ratio of 65:35 (*R*):(*S*), on average) [12]. Sensory analysis (gas chromatography–olfactometry) found that γ-nonalactone is strongly associated with prune aroma in aged red wines, and is hypothesised to be a contributor to undesirable prune aroma in prematurely aged red wines, along with another aroma compound, 3-methyl-2,4-nonanedione [4].

**Scheme 1:**
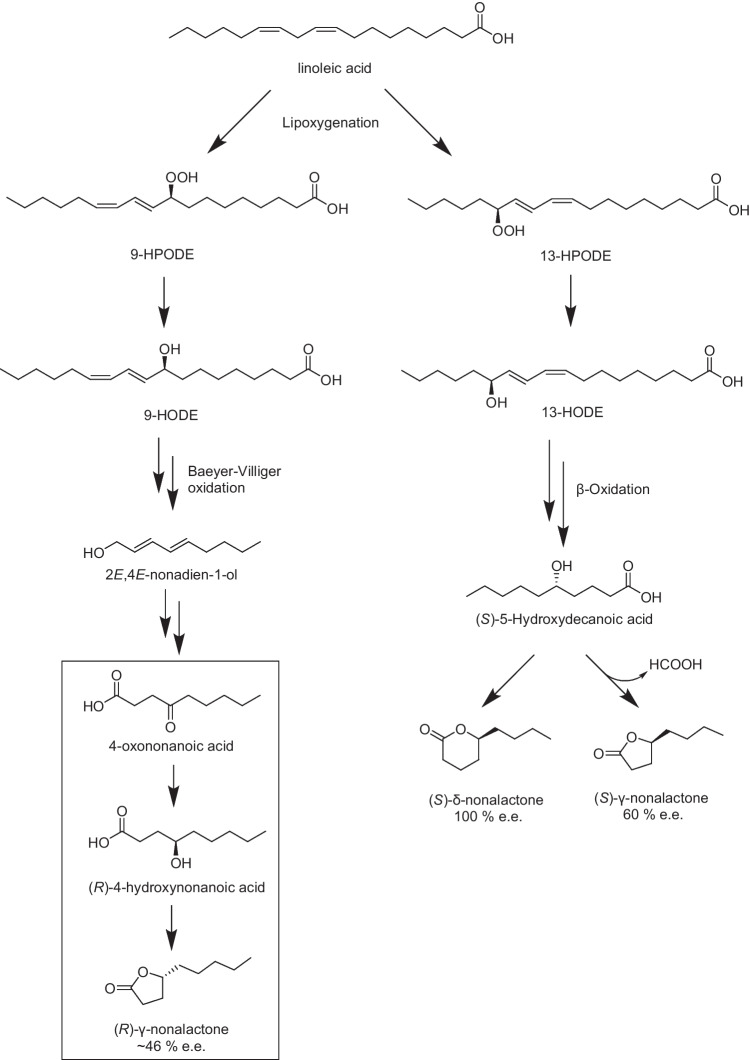
Proposed route of γ-nonalactone **1** biogenesis from linoleic acid, via 9-HODE and 13-HODE [13]. Hypothesised conversion of 4-oxononanoic acid to (*R*)-γ-nonalactone also included (portion of scheme in box) [12]

The route(s) of biogenesis of γ-nonalactone in grapes and/or wine have only been partially elucidated [2]. Through ^18^O-labelling experiments in beer, linoleic acid and its 9- and 13-lipoxygenation products 9-hydroxydecadienoic acid (9-HODE) and 13-hydroxydecadienoic acid (13-HODE) were found to be putative precursors to γ-nonalactone during fermentation (Scheme 1) [13]. In another work, grape musts were spiked with ^2^H-labelled 4-oxononanoic acid prior to fermentation, and analysis of the resulting wine showed that ^2^H-labelled γ-nonalactone had been produced, confirming 4-oxononanoic acid as a precursor. This result suggests that the ketone functional group of 4-oxononanoic acid undergoes reduction to an alcohol. The resulting 4-hydroxynonanoic acid can then undergo lactone ring-closure, which is thermodynamically favourable under acidic conditions, as found in wine [12]. Despite these key findings, it is not clear how 4-oxononanoic acid is formed, nor how lipoxygenation may be occurring (Scheme 1). Consequently, further work is needed to clarify the formation pathway(s) of γ-nonalactone.

Due to the complex nature of the chiral analysis, most analyses tend to treat these lactones as a racemic mixture [2, 3, 13]. The complexity of the wine matrix means that sophisticated techniques are required for the accurate and precise quantification of specific aroma compounds, particularly those found at low concentrations, including γ-nonalactone. Extraction of γ-nonalactone, along with other low-polarity aroma compounds from the wine matrix, has most commonly been carried out via solid-phase extraction (SPE) [3, 14], headspace solid-phase microextraction (HS-SPME) [11, 15, 16], liquid–liquid extraction (LLE) [9], stir-bar sorptive extraction (SBSE) [17], or variations of these methods [2]. SPE generally involves passing the sample through a cartridge containing solid material, to which the analytes (in this case low-polarity aroma compounds) adsorb. Washing steps remove undesirable interferents in the matrix, while analytes are retained in the solid phase. Finally, a solvent in which the analytes are highly soluble is passed through the cartridge, eluting the analytes. Due to the volatility of γ-nonalactone, GC–MS is the most common means of separation and quantification of this compound [2].

Several internal standards have been used for semi-quantitative or quantitative analysis of γ-nonalactone in wines, including 2-octanol [13, 16], 4-methyl-2-pentanol [9], and 3,3-dimethylphenol [17]. However, for quantitative analysis, an isotopologue of the analyte of interest is the most ideal internal standard for a stable isotope dilution assay (SIDA). SIDA was used for the quantification of saturated linear aliphatic lactones in a range of Australian wines, including γ-octalactone, γ-nonalactone, γ-decalactone, and γ-dodecalactone. ^2^H_7_-analogues of these compounds were synthesised, which were used as internal standards during extraction and quantification, using SPE and subsequent analysis through GC–MS [3]. A similar deuterium exchange step was used for the synthesis of ^2^H_7_-γ-decalactone, which was subsequently used as an internal standard for the quantification of several linear aliphatic lactones (γ- and δ-nona- to dodecalactones), instead using HS-SPME for sample extraction [18].

In this investigation, when using the aforementioned ^2^H-labelling route, incomplete deuterium exchange was observed. This observation led to the synthesis of a novel ^2^H_2_^13^C_2_-labelled γ-nonalactone standard in this work, using Wittig olefination and deuterogenation steps for the introduction of ^13^C and ^2^H atoms into the molecule, respectively. This novel standard was subsequently used for the robust analysis of 12 commercial New Zealand (NZ) Pinot noir wines via SIDA, using an established SPE-GC–MS method for the accurate and sensitive quantification of γ-nonalactone. Since γ-nonalactone has been measured in concentrations well above its ODT (up to 155 µg L^−1^) in Pinot noir wines from Oregon, USA [17], this work also aims to determine whether γ-nonalactone may have an important role in the aroma profile of NZ Pinot noir wines for the first time.

## Materials and methods

### Chemicals and reference compounds

γ-Nonalactone (> 97%), isopropyl bromide, oxalyl chloride (> 98%), diisobutylaluminium hydride (1.0 M in toluene), deuterium chloride (35% in ^2^H_2_O), sodium borohydride (98%), heptaldehyde (95%), selenium dioxide, deuterium gas, and hydrobromic acid (48%, aq.) were purchased from Sigma-Aldrich (MO, USA). Benzoic acid (AR), triphenylphosphine (97%), and Pd on activated carbon (10%) were purchased from AK Scientific (CA, USA). ^13^C_2_-bromoacetic acid, deuterium oxide, and deuterochloroform were purchased from Cambridge Isotope Laboratories (MA, USA). Magnesium sulfate, sodium hydroxide, potassium hydroxide, tartaric acid, and all organic solvents were purchased from ECP Ltd (Auckland, New Zealand). A Sartorius (Göttingen, Germany) Arium Pro Ultrapure Water System was used for Type 1 water production. Chemicals were used as purchased, unless otherwise specified.

### General synthetic details

Thin-layer chromatography (TLC) was performed using Merck silica gel 60 F_254_ aluminium sheets. Short-wave ultraviolet (UV) fluorescence and vanillin and potassium permanganate (KMnO_4_) stains were used for TLC spot visualisation. Flash chromatography was performed using Chem-Supply silica gel 60 (particle size 0.04–0.06 mm).

Proton (^1^H) and carbon (^13^C) NMR were recorded using a Bruker AVIII400 spectrometer (MA, USA), at frequencies of 400 and 100 MHz, respectively. Deuterochloroform (C^2^HCl_3_) or deuterium oxide (^2^H_2_O) were used as sample solvents and internal references. ^1^H NMR data were reported as follows: chemical shift (δ, ppm), relative integral, multiplicity (s = singlet, d = doublet, t = triplet, q = quartet, qn = quintet, dd = doublet of doublets, dq = doublet of quartets, ddquin = doublet of doublet of quintets, qd = quartet of doublets, ddq = doublet of doublet of quartets, sept = septet). High-resolution mass spectra (HRMS) were recorded using a Bruker MicrOToF-QII mass spectrometer, coupled to an electrospray ionisation source (ESI), and analysis performed in positive ionisation mode. Infrared (IR) spectra were recorded using a PerkinElmer (MA, USA) FT-IR Spectrum Two Spectrophotometer, equipped with a UATR Two attachment.

### General analytical details

Bond Elut-ENV 200 mg and 3 mL SPE cartridges were purchased from Agilent Technologies (CA, USA). Analyses of wine samples were conducted using an Agilent Technologies 6890N Network GC coupled to a Single Quadrupole Agilent Technologies 5973 inert mass selective detector. SCAN mode was used for all samples. The GC column used was Agilent Technologies HP-INNOWax (polyethylene glycol stationary phase, 60 m length, 0.25 mm inner diameter, 0.25 µm film thickness, and temperature limits 40–260 °C). Samples (1 µL) were injected with an Agilent Technologies 7683B Series Splitless Injector. The carrier gas used was He, with a flow rate of 1 mL min^−1^. A temperature gradient was utilised for all samples; beginning at 50 °C, the temperature was increased to 60°C at a rate of 1°C min^−1^, then to 250°C at a rate of 10°C min^−1^, after which it was held at 25°C for 25 min, for a total run time of 54 min.

Alcohol levels (v/v) in commercial wine samples were analysed using an Alcolyzer Wine M from Anton Paar (Graz, Austria). pH and titratable acidity (TA) of commercial wine samples were determined using a Hanna Instruments (RI, USA) Wine Titrator, equipped with a Hanna Instruments pH meter. Residual sugar (RS) and free and total sulfite levels were determined in commercial wine samples using the Megazyme (Bray, Ireland) D-Fructose/D-Glucose Assay Kit and Total and Free Sulfite Assay Kit, respectively.

### Wine samples and model wine

Dry red cask wine for calibration curve construction and 12 commercial NZ Pinot noir wines were purchased from retailers in Auckland, NZ. Cask wine was non-vintage with unspecified grape varieties. Pinot noir wines were selected from three key NZ Pinot noir–producing regions (Central Otago, Marlborough, and Waipara Valley), with a range of recent vintages (2018, 2019, 2020, and 2021) and a wide price range (NZ$18.99–NZ$99.99), to provide a representative set of NZ Pinot noir wines. Measured technical data for these wines are given in Table S1. Model wine was comprised of tartaric acid (5 g) dissolved in a solution of 12% (v/v) ethanol in type 1 water (1 L), with the pH adjusted to 3.2 using NaOH (*aq.*, 3 M then 1 M).

### ^2^H_6_-γ-Nonalactone synthesis

**Scheme 2:**
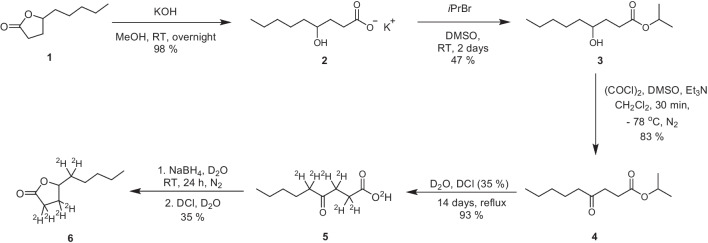
^1^H-^2^H exchange of α-protons of isopropyl 4-oxononanoate **4** during the synthesis of ^2^H_6_-γ-nonalactone **6**. In this work, NaBH_4_ was used instead of NaB^2^H_4_ for the reduction of the ketone group [3]

Synthesis of a deuterated (^2^H_6_) internal standard was initially attempted, based on the synthesis of ^2^H_7_-γ-nonalactone by Cooke et al. [3]. The scale was reduced from that used in literature, and in the final step, NaBH_4_ was used as a reducing agent instead of NaB^2^H_4_, resulting in a ^2^H_6_-labelled γ-nonalactone analogue rather than a ^2^H_7_-labelled analogue (Scheme 2 and see SI for further details of this investigation). Despite hydrogen–deuterium exchange being carried out under reported conditions (reflux in ^2^HCl/^2^H_2_O), and extraction, then replacement of the ^2^HCl/^2^H_2_O ^1^H-^2^H exchange mixture after 7 days, and reaction for a further 7 days, complete ^1^H-^2^H exchange was not observed and thus the deuterated internal standard was not deemed appropriate for use for accurate quantitative analysis.

### ^2^H_2_^13^C_2_-γ-Nonalactone synthesis

^*13*^*C*_*2*_*-Ethyl bromoacetate* **8:** to a solution of ^13^C_2_-bromoacetic acid **7** (0.33 g, 2.34 mmol) in dry ethanol (1.5 mL), under an atmosphere of nitrogen, H_2_SO_4_ (conc., 2 drops) was added, and the resulting mixture was heated at 60 °C for 6 h, with stirring. The mixture was then cooled to room temperature, and water (10 mL) was added. The mixture was extracted with CH_2_Cl_2_ (3 × 10 mL portions); then, the combined organic extracts were dried (MgSO_4_) and concentrated in vacuo to give ^13^C_2_-ethyl bromoacetate **8** as a colourless oil (0.396 g, quantitative), which was used in the next reaction without further purification.

- ^**1**^**H NMR (400 MHz, C**^**2**^**HCl**_**3**_**)** 1.30 (t, 3H, H-2′, *J* = 7.1 Hz), 3.82 (dd, 2H, H-2, *J* = 4.6, 153.2 Hz), 4.24 (dq, 2H, H-1′, *J* = 3.1, 7.1 Hz). ^1^H NMR was in agreement with literature values [19, 20].

^*13*^*C*_*2*_*-(Ethoxycarbonylmethyl)triphenylphosphonium bromide* **9**: a solution of ^13^C_2_-ethyl bromoacetate **8** (0.390 g, 2.31 mmol) in ethyl acetate (2 mL) was slowly added to a solution of triphenylphosphine (0.700 g, 2.67 mmol) in ethyl acetate (2 mL) and the mixture stirred overnight at room temperature. The resulting white precipitate was filtered, washed with diethyl ether (3 × 2 mL portions), and dried in vacuo to give ^13^C_2_-(ethoxycarbonylmethyl)triphenylphosphonium bromide **9** (0.909 g, 91%) as a white crystalline powder, which was used in the next reaction without further purification.

- ^**1**^**H NMR (400 MHz, C**^**2**^**HCl**_**3**_**)** 1.08 (t, 3H, H-2′, *J* = 7.1 Hz), 4.05 (dq, 2H, H-1′, *J* = 3.1, 7.1 Hz), 5.39–5.86 (m, 2H, H-2), 7.64–7.72 (m, 6H, H-Ar), 7.75–7.82 (m, 3H, H-Ar), 7.87–7.96 (m, 6H, H-Ar). ^1^H NMR was in agreement with literature values [21].

^*13*^*C*_*2*_*-(Carbethoxymethylene)triphenylphosphorane* **10**: to a solution of ^13^C_2_-(ethoxycarbonylmethyl)triphenylphosphonium bromide **9** (0.900 g, 2.09 mmol) in CH_2_Cl_2_ (5 mL) NaOH (*aq.*, 1.0 M, 5 mL) was added and the mixture stirred vigorously for 15 min. The organic layer was collected, and the aqueous layer was extracted with CH_2_Cl_2_ (3 × 5 mL portions). The combined organic extracts were dried (MgSO_4_) and concentrated in vacuo to give ^13^C_2_-(carbethoxymethylene)triphenylphosphorane **10** (0.722 g, 99%) as a white powder which was used in the next reaction without further purification.

- ^**1**^**H NMR (400 MHz, C**^**2**^**HCl**_**3**_**)** 1.04 (m, H-2′), 3.96 (m, 2H, H-1′), 7.41–7.49 (m, 6H, H-Ar), 7.51–7.58 (m, 3H, H-Ar), 7.61–7.70 (m, 6H, H-Ar). ^1^H NMR was in agreement with literature values [21].

^*13*^*C*_*2*_*-Ethyl non-2-enoate* **12**: to a solution of heptaldehyde **11** (0.312 g, 0.422 mL, 2.73 mmol) in CH_2_Cl_2_ (5 mL) was added a solution of ^13^C_2_-(carbethoxymethylene)triphenylphosphorane **10** (1.00 g, 2.85 mmol) in CH_2_Cl_2_ (5 mL), and the mixture was stirred under an atmosphere of nitrogen, at room temperature, overnight. The solvent was removed in vacuo and pentane (10 mL) was added. The resulting suspension was stirred for 1 h, then filtered through a short pad of Celite. The solvent was removed in vacuo to give the crude product, which was purified by flash chromatography (98:2 pentane/Et_2_O) to give ^13^C_2_-ethyl non-2-enoate **12** (0.447 g, 88%) as a colourless oil.

- **R**_**f**_** (98:2 pentane/Et**_**2**_**O)** 0.32
- ^**1**^**H NMR (400 MHz, C**^**2**^**HCl**_**3**_**)** 0.88 (t, 3H, H-9, *J* = 7.0 Hz), 1.29 (t, 3H, H-2′, *J* = 7.1 Hz), 1.31–1.37 (m, 2H, H-5), 1.40–1.50 (m, 6H, H-6, H-7, H-8), 2.14–2.24 (m, 2H, H-4), 4.18 (dq, 2H, H-1′, *J* = 3.0, 4.1 Hz), 5.81 (ddquin, 1H, H-2, *J* = 1.6, 15.6, 161.6 Hz), 6.91–7.01 (m, 1H, H-3))
- ^**13**^**C NMR (100 MHz, C**^**2**^**HCl**_**3**_**)** 14.1 (C-9), 14.3 (C-2′), 22.6 (C-4, C-5, C-6, or C-7), 22.7(C-4, C-5, C-6, or C-7), 29.0 (C-4, C-5, C-6, or C-7), 29.1 (C-4, C-5, C-6, or C-7), 31.7 (C-4, C-5, C-6, or C-7), 59.8 (C-1′), 119.6 (d, C-2), 150.6 (C-3), 166.6 (d, C-1)
- **IR**: ***υ***_**max**_** (film)/cm**^**−1**^ 2958, 2928, 2858, 1677, 1626, 1231, 1153, 975
- ***m/z (ESI***^**+**^**)** 209 (MNa^+^, 40%), 187 (MH.^+^, 100), 159 (99), 149 (4), 141 (6)
- **HRMS** Found (Mna^+^): 209.1412, expect 209.1409 for ^13^C_2_C_9_H_20_O_2_Na^+^

^*13*^*C*_*2*_*-Ethyl 4-hydroxy-non-2-enoate* **13**: to a solution of ^13^C_2_-ethyl non-2-enoate **12** (0.43 g, 2.31 mmol) in a mixture of dioxane (3 mL) and water (0.33 mL) selenium dioxide (0.52 g, 4.69 mmol) was added, and the resulting mixture was heated at reflux overnight, under an atmosphere of nitrogen. The mixture was then cooled to room temperature, and water (3 mL) was added. The resulting solid was filtered, then the filtrate was extracted with CH_2_Cl_2_ (3 × 10 mL portions). The combined organic layers were dried (MgSO_4_) and concentrated in vacuo to give the crude product, which was subsequently purified by flash chromatography (85:15 pentane/EtOAc) to give ^13^C_2_-ethyl 4-hydroxy-non-2-enoate **13** (0.170 g, 36%) as a pale-yellow oil.

- **R**_**f**_** (85:5 pentane/EtOAc)** 0.39
- ^**1**^**H NMR (400 MHz, C**^**2**^**HCl**_**3**_**)** 0.88 (t, 3H, H-9, *J* = 6.7 Hz), 1.25–1.67 (m, 11H, H-2′, H-5, H-6, H-7, and H-8), 4.20 (qd, 2H, H-1′, *J* = 3.1, 7.2 Hz), 4.27–4.35 (m, 1H, H-4), 6.02 (ddq, 1H, H-2, J_H-C_ = 163.4 Hz, *J* = 1.6, 15.7 Hz), 6.90–6.99 (m, 1H, H-3)
- ^**13**^**C NMR (100 MHz, C**^**2**^**HCl**_**3**_**)** 14.1 (C-9), 14.2 (C-2′) 21.1 (C-5, C-6, C-7, or C-8), 22.8 (C-5, C-6, C-7, or C-8), 26.0 (C-5, C-6, C-7, or C-8), 31.6 (C-5, C-6, C-7, or C-8), 35.2 (C-5, C-6, C-7, or C-8), 60.4 (C-1′), 67.1 (C-4), 120.2 (d, C-2, *J*_C-C_ = 119.8 Hz), 145.7 (C-3), 166.5 (d, C-1, *J*_C-C_ = 75.2 Hz)
- **IR**: *υ*_max_ (film)/cm^−1^ 3441, 2957, 2860, 1677, 1661, 1242, 1151, 1035, 979
- ***m/z (ESI***^**+**^**)** 225 (MNa^+^, 100%), 199 (10), 153 (10), 137 (9), 121 (8)
- **HRMS** Found (MNa^+^) 225.1365, expect 225.1358 for ^13^C_2_C_9_H_20_O_3_Na^+^

^*2*^*H*_*2*_^*13*^*C*_*2*_*-Ethyl 4-hydroxynonanoate* **14:** to a solution of ^13^C_2_-ethyl 4-hydroxy-non-2-enoate **13** (0.160 g, 0.79 mmol) in ethanol (10 mL), palladium on activated carbon (10% loading, 20 mg) was added. This mixture was placed under an atmosphere of deuterium gas (99.7% purity) and stirred for 2 h, at room temperature. The mixture was then filtered through a short pad of Celite, which was then washed with ethanol (2 × 50 mL portions). The solvent was removed in vacuo to give ^2^H_2_-^13^C_2_-ethyl 4-hydroxynonanoate **14** (0.120 g, 74%) as a pale-yellow oil, which was used in the following reaction without further purification.

^*2*^*H*_*2*_^*13*^*C*_*2*_*-γ-Nonalactone (*^*2*^*H*_*2*_*-*^*13*^*C*_*2*_*-pentyldihydrofuran-2(3H)-one)* **15:** to a solution of ^2^H_2_^13^C_2_-ethyl 4-hydroxynonanoate **14** (0.120 g, 0.58 mmol) in a mixture of THF (2 mL) and water (1 mL) was added NaOH (0.13 g, 3.25 mmol). The resulting mixture was stirred for 2 h, then HCl (1.0 M) was added dropwise until the pH was 1. The mixture was then extracted with CH_2_Cl_2_ (3 × 5 mL portions). The combined organic extracts were dried (Na_2_SO_4_) and the solvent removed in vacuo to give the crude product, which was purified by flash chromatography (85:15 pentane/EtOAc) to give ^2^H_2_^13^C_2_-γ-nonalactone **15** (0.035 g, 38%) as a colourless oil.

- **R**_**f**_** (85:15 pentane/EtOAc)** 0.45
- ^**1**^**H NMR (400 MHz, C**^**2**^**HCl**_**3**_**)** 0.89 (t, 3H, H-9, *J* = 6.9 Hz), 1.22–1.51 (m, 6H, H-6, H-7, and H-8), 1.54–1.64 (m, 2H, H-5), 1.68–1.78 (m, 1.23*, H-5), 1.79–1.89 (m, 0.72H*, H-2, and/or H-3) 2.26–2.38 (m, 1.28H*, H-2 and/or H-3), 2.62–2.72 (m, 0.75H*, H-2, and/or H-3) 4.44–4.52 (m, 1H, H-4)
- ^**13**^**C NMR (100 MHz, C**^**2**^**HCl**_**3**_**)** 14.0 (C-9), 22.5 (C-5, C-6, C-7, or C-8), 24.9 (C-5, C-6, C-7, or C-8), 27.9–29.3 (m, C-2, and C-3), 31.6 (C-5, C-6, C-7, or C-8), 35.6 (C-5, C-6, C-7, or C-8), 81.1 (C-4), 177.3 (d, C-1, *J*_C-C_ = 49.0)
- **IR**: *υ*_max_ (film)/cm^−1^ 2955, 2931, 2860, 1724, 1460, 1157, 1114, 947
- ***m/z (ESI***^**+**^**)** 199 (5%), 183 (MNa^+^, 100), 143 (5)
- **HRMS** Found (MNa^+^) 183.1096, expect 183.1242 for ^13^C_2_C_7_^2^H_2_H_14_O_2_Na^+^
*Non-integer integrals (approximations) are provided where deuterium labelling has been used.

### Sample preparation: solid-phase extraction

SPE cartridges (Bond Elut-ENV 200 mg, 3 mL) were conditioned with methanol (2 mL), then water (4 mL). Wine samples (50 mL) spiked with internal standard **15** (100 µL of 10,000 µg L^−1^ in ethanol) were then passed through the SPE cartridges, which were subsequently washed with water (5 mL), then a solution of methanol (40% v/v) and sodium bicarbonate in type 1 water (1% w/v, 20 mL). The cartridges were allowed to dry for 30 min, by passing air through them. Finally, analytes were eluted using CH_2_Cl_2_ (2.5 mL, giving a sample enrichment factor of 20), which was collected and dried with Na_2_SO_4_, before being filtered and concentrated at 30 °C under a stream of nitrogen, to an approximate final volume of 100 µL, before analysis by GC–MS [15].

### Verification of suitable SIDA internal standard

The stability of ^2^H_2_^13^C_2_-γ-nonalactone **15** during spiking and extraction was verified by comparing more standard spiking conditions to more elevated conditions (higher temperatures and/or longer time periods) and determining whether any changes in relative abundances of isotopologues were observed. Model wine samples (50 mL) were spiked with internal standard (100 µL of 10,000 µg L^−1^ in ethanol). After spiking, the samples were subjected to the following conditions, in triplicate: 30 min at room temperature (standard conditions), and 30 min at 40 °C, 3 h at room temperature or 3 h at 40 °C (extreme conditions). Subsequently, extraction and analysis by SPE-GC–MS were conducted as stated above. Additionally, samples of ^2^H_2_^13^C_2_-γ-nonalactone dissolved in CH_2_Cl_2_ were analysed by GC–MS, for comparison. Samples were compared to ^2^H_2_^13^C_2_-γ-nonalactone **15** dissolved in CH_2_Cl_2_, by observing the relative peak areas of isotopically labelled γ-nonalactone **15** (base peak *m/z* 89), and the peaks which represented the loss of one or two mass units (*m/z* 88 and 87), which would correspond to the exchange of one or two deuterium atoms with hydrogen atoms, respectively.

### Calibration curves and method validation

Calibration curves were constructed in two different matrices: dry red cask wine and model wine. For each matrix, 50 mL samples were spiked with internal standard (100 µL of 10,000 µg L^−1 2^H_2_^13^C_2_-γ-nonalactone in ethanol) to give a concentration of 20 µg L^−1^, and a range of volumes of unlabelled γ-nonalactone (10,000 µg L^−1^ in ethanol) to give concentrations of 0, 0.5, 1, 2, 5, 10, 25, 50, and 100 µg L^−1^, each in triplicate, while 5 µg L^−1^ and 25 µg L^−1^ were each repeated seven times for initial assessment of the repeatability of this method. This data was included in the final calibration curves for completeness, but further repeatability and reproducibility assessments were carried out, as described below. Calibration samples were extracted and analysed as described above, using SPE-GC–MS. The order of analysis was randomised.γ-Nonalactone **1** and ^2^H_2_^13^C_2_-γ-nonalactone **15** had retention times of 27.96 and 27.94 min, respectively (Fig. S1). Relative responses of γ-nonalactone **1** and ^2^H_2_^13^C_2_-γ-nonalactone **15** were measured using areas of their respective base peaks; *m/z* 85 and 89. The hypothesised fragmentation pathway from their respective parent ions is shown in Scheme S1. Qualifier ions used were *m/z* 99 and 114 for γ-nonalactone, and *m/z* 103 and 118 for ^2^H_2_^13^C_2_-γ-nonalactone **15**. Calibration curves were constructed by plotting relative responses of γ-nonalactone and ^2^H_2_^13^C_2_-γ-nonalactone **15** against known concentrations of γ-nonalactone **1**. In the dry red wine calibration, the relative response at 0 µg L^−1^ added γ-nonalactone **1** was significantly higher than expected, indicating that γ-nonalactone **1** was already present in the matrix. Standard addition analysis revealed a γ-nonalactone **1** concentration of 12 µg L^−1^ in the dry red wine matrix. It was therefore necessary to further verify the relationship between relative response and γ-nonalactone **1** concentration, particularly below 12 µg L^−1^, to avoid extrapolation when analysing unknown samples if possible. Model wine was selected for this purpose, as it was known that no γ-nonalactone **1** was already present in this matrix. The resulting calibration curve was constructed in the same manner as for the dry red wine. Calibration curve slopes were shown not to be statistically different at the 95% confidence level (see SI for details of the calibration curves, including the 95% confidence interval for the slope estimates, showing that they overlap). Thus, it could be concluded that the model wine calibration curve accurately reflected the relationship between γ-nonalactone **1** concentration and the relative response of γ-nonalactone **1** and the internal standard **15** within the dry red wine matrix and was therefore used for quantification in the Pinot noir wines.

In order to verify the repeatability and reproducibility of this analytical method, instrumental and experimental repeats were carried out, respectively, according to recommended analytical best practice [22]. Instrumental repeats were carried out using one model wine sample spiked with 5 µg L^−1^ γ-nonalactone **1** and 10 µg L^−1 2^H_2_^13^C_2_-γ-nonalactone **15**, and repeating the sample analysis seven times in as short a period of time as possible, i.e. consecutive analyses of the sample within the same day. Experimental repeats were conducted with the same concentrations of analyte and internal standard in model wine, with the full experimental method (sample preparation and extraction) being repeated in triplicate, on five consecutive days. Instrumental and experimental repeatability metrics for the method were obtained; 0.38 and 0.72%, respectively). Percentage recovery was calculated using the experimental repeats and was 104%.

Finally, the limit of detection (LOD) and limit of quantitation (LOQ) were calculated for the model wine calibration curve, using the following formulae (where *a* = estimate of the *y* intercept and *s* = standard error of the calibration curve) [23]:

$$LOD=a+3.29s$$

$$LOQ=a+10s$$

Values for LOD and LOQ were 0.4 and 1.1 µg L^−1^, respectively.

### Quantification of γ-nonalactone in 12 NZ Pinot noir samples

Samples of 12 commercial NZ Pinot noir wines (50 mL) were spiked with ^2^H_2_^13^C_2_-γ-nonalactone **15** (100 µL of 10,000 µg L^−1^ in ethanol solution), each in triplicate. Samples were extracted and analysed according to the SPE-GC–MS method described above. The order of sample analysis was randomised. The relative responses of γ-nonalactone **1** and ^2^H_2_^13^C_2_-γ-nonalactone **15** were used to calculate the concentrations of γ-nonalactone **1** present in each sample using the calibration curve and stated γ-nonalactone **1** concentrations calculated for each wine were the average of the three experimental replicates. Any outliers were detected using Grubbs’ test, and subsequently removed.

Relationships between the variables of vintage, region of production, and price with γ-nonalactone **1** concentration for each wine were investigated. To assess if there were any significant effects from any of the factors, multiple linear regression (stats package) was used [24]. R software was used for data analysis, and the ggplot2 package in R was used to visualise the data.

## Results and discussion

SIDA was selected for quantification of γ-nonalactone **1** in wine samples, as isotopically labelled internal standards are expected to behave in an equivalent way as their natural analogues during sample extraction and analysis. Since a method for SIDA of γ-nonalactone **1** in wine was already established [3], synthesis of ^2^H_6_-γ-nonalactone was initially attempted, adapting the reported method. Starting with commercially available unlabelled γ-nonalactone **1**, lactone ring opening, followed by isopropyl ester formation, was carried out. Next, Swern oxidation of the 4-hydroxyl group was performed, to give isopropyl 4-oxononanoate **4**. Regrettably, problems were encountered during the deuterium labelling step. Isopropyl 4-oxononanoate **4** was heated at reflux with deuterium oxide (^2^H_2_O) and deuterium chloride (35% w/v in ^2^H_2_O) for 7 days, following the literature procedure (Scheme 2) [3]. The reaction mixture was then extracted with diethyl ether, and the product, 4-oxononanoic acid **5**, was obtained and characterised via ^1^H NMR and HRMS. Mixtures of deuterium-labelled isotopologues of this compound were observed, evidenced by peaks remaining in the ^1^H NMR spectrum corresponding to protons which should have been completely replaced by deuterium atoms. This result was corroborated by the HRMS spectrum, with mixtures of *m/z* base peaks observed, indicating predominance of ^2^H_4_- and ^2^H_5_-4-oxononanoic acid isotopologues, rather than the intended ^2^H_6_-4-oxononanoic acid **5**.

Consequently, heating of this compound at reflux in ^2^HCl/^2^H_2_O was continued for a further 7 days, before extraction and characterisation were repeated. A mixture of isotopologues was again observed via ^1^H NMR and HRMS (Figs. S2 and S3). No mention of this phenomenon was given by the authors; however, a similar deuterium exchange step, using isopropyl 4-oxodecanoate, was carried out in another work, for the synthesis of a deuterated γ-decalactone analogue. The final deuterated γ-decalactone analogue was reported to have reduced deuterium incorporation, and purity of ^2^H_7_-γ-decalactone was 70%, relative to a pure sample of unlabelled γ-decalactone [18]. Despite reportedly successful quantification of desired lactones in wine by Cooke et al. [3] and Langen et al. [18], this mixture of isotopologues was considered unacceptable for the purposes of SIDA in this work; thus, the synthesis of an alternative isotopically labelled internal standard was pursued for the quantification of γ-nonalactone **1**.

**Scheme 3:**
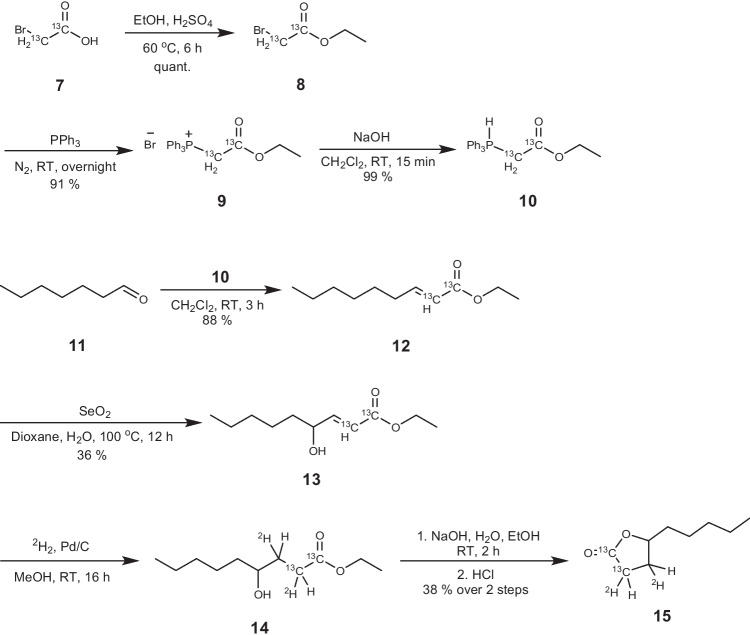
Synthesis of ^13^C_2_-^2^H_2_-γ-nonalactone **15** in this work based on a previously reported synthetic route [25]

Labelling of the lactone ring, rather than the alkyl chain, was desirable as during ionisation, the major fragmentation product from γ-nonalactone **1** results from the loss of the alkyl chain (Scheme S1). It was decided to follow a literature method for the synthesis of ^13^C_2_-γ-nonalactone [25], and then adapt it to introduce two ^2^H atoms, to give a difference in *m/z*- ratio of 4 units between the natural analogue and the isotopically labelled internal standard. Scheme 3 shows the reaction pathway employed for the synthesis of ^13^C_2_-^2^H_2_-γ-nonalactone **15**. For the introduction of two ^13^C atoms, a single Wittig olefination step was utilised. A stabilised Wittig reagent **10** was synthesised from commercially available ^13^C_2_-bromoacetic acid **7** over three steps [19]*.* Subsequently, Wittig olefination was carried out with commercially available heptaldehyde **11**. A hydroxyl group was then introduced at the 4-position of **12** via allylic hydroxylation, using SeO_2_. Deuterogenation was used to saturate the double bond in **13** using ^2^H_2_ gas. Finally, the ester group in **13** was hydrolysed to liberate the 4-hydroxyacid, which underwent thermodynamically favourable lactone ring-closure under acidic conditions to give the desired target ^13^C_2_-^2^H_2_-γ-nonalactone **15**.

As this isotopically labelled standard **15** is novel, it was necessary to ensure that it is stable during the sample extraction and analysis processes and thus generally suitable for use as an internal standard. Previous literature has reported the occurrence of hydrogen–deuterium scrambling during deuterogenation reactions (hydrogenation using deuterium gas), due to the mechanism of the reaction as it interacts with the palladium catalyst [26]. This phenomenon was observed in this work, with the product of the deuterogenation reaction (^2^H_2_^13^C_2_-ethyl 4-hydroxynonanoate **13**) being incompletely labelled, as demonstrated by analysis via both ^1^H NMR and HRMS. This was not ideal, as a small portion of the internal standard would not have the expected mass-to-charge ratio during analysis by GC–MS. However, if it could be demonstrated that the levels of the different internal standard isotopologues were consistent between samples, the fragmentation of the internal standard was consistent, and the internal standard isotopologues did not interfere with the mass-to-charge ratio of the analyte being monitored; this could still be an appropriate internal standard for SIDA. Additionally, the presence of a deuterium atom adjacent to a carbonyl group meant that deuterium exchange could be possible in acidic media, such as wine, which would decrease the mass-to-charge ratio of the internal standard; thus, it would not fulfil the requirement of being stable for SIDA. It was therefore necessary to ensure that no significant deuterium exchange was occurring.

The stability of the internal standard **15** was evaluated by replicating the spiking method in model wine at more elevated conditions (high temperature of 40 °C, long period of time in wine matrix of 3 h) compared to the more standard conditions (room temperature, 30 min in wine matrix). Each condition was trialled in triplicate. Samples were subsequently extracted following the typical SPE procedure, then analysed by GC–MS. The relative intensities corresponding to the base peak of ^2^H_2_^13^C_2_-γ-nonalactone (*m/z* 89) and its partially labelled isotopologues ^2^H_1_^13^C_2_-γ-nonalactone (*m/z* 88) and ^13^C_2_-γ-nonalactone (*m/z* 87) were calculated for each of the samples. No significant trends were observed in the relative peak areas of these isotopologues, due to neither time nor temperature (Table S2). Consequently, ^2^H_2_^13^C_2_-γ-nonalactone **15** was considered sufficiently stable as an internal standard under standard spiking conditions. For quantification of the analyte in wine samples, the peak area of ^2^H_2_^13^C_2_-γ-nonalactone **15** (*m/z* 89) only was used in calculations, as this isotopologue was shown via mass spectrometry to be the most abundant.

Initially, dry red cask wine was used as the calibration matrix, to closely mimic the matrices of the samples to be analysed. Known concentrations of γ-nonalactone **1** (ranging from 0 to 100 µg L^−1^) and internal standard **15** (10 µg L^−1^) were used to spike the red wine. The resulting calibration curve had excellent linearity (*R*^2^ = 0.997) (Fig. S4, Table S3). However, the intercept of the linear regression showed that the red wine matrix already had γ-nonalactone **1** present (12.0 µg L^−1^), prior to spiking. This was undesirable, as it could not be determined that the relationship between the relative response of γ-nonalactone **1** and the internal standard **15** remained linear below the concentration of γ-nonalactone **1** present in the red wine. Thus, an additional calibration curve was constructed in an identical manner, using model wine. Although this matrix does not very accurately resemble that of the samples to be analysed, it was known that no γ-nonalactone **1** was present in the matrix prior to spiking. As SIDA was being used, it was also anticipated that the matrix should not significantly impact the relative responses of γ-nonalactone **1** and the internal standard.

**Fig. 2:**
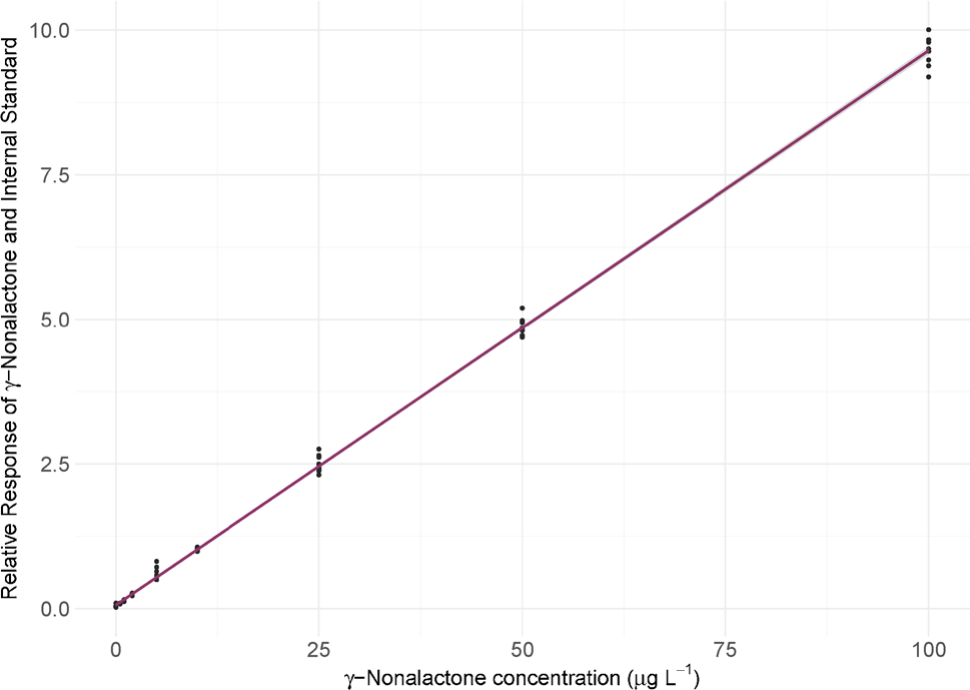
Model wine calibration curve showing relative peak areas of γ-nonalactone and ^2^H_2_^13^C_2_-γ-nonalactone **15** with changing concentrations of γ-nonalactone 1 (µg L^−1^)

The resulting model wine calibration curve (Fig. 2) also had excellent linearity (*R*^2^ = 0.9985) (Table S4). It was determined that at the 95% confidence level, the gradients of the red wine and model wine calibration curves were not different; thus, the model wine calibration curve was deemed appropriate for NZ Pinot noir wine analysis. Experimental repeatability was assessed by repeating model wine spiking (5 µg L^−1^) and SPE-GC–MS extraction and analysis in triplicate, on five different days. Similarly, the reproducibility of the GC–MS instrument was assessed by analysing the same sample (5 µg L^−1^) seven consecutive times [22]. Repeatability and reproducibility were found to be 0.72% and 0.38% (Tables S5 and S6), respectively. The LOD and LOQ were 0.4 and 1.1 µg L^−1^, respectively (Table S4), which were also considered appropriate for analysis, as they are well below the ODT of γ-nonalactone. These values are similar to those reported in previous literature for the quantification of γ-nonalactone **1** in wine (Table 1) [3, 5, 15, 16, 18, 27-30]. The percentage recovery of γ-nonalactone **1** for this method was estimated at 104%, which is either on par with or an improvement on previous methods [15].

**Table 1:** Summary of the methods used previously for quantification of γ-nonalactone **1** in wine, and their associated LOD (µg L^−1^)

Year	Internal standard	LOD (µg L^−1^)	Extraction method	Analysis method	Reference
1998	2-Octanol	0.38	Demixing/microextraction	GC-ion trap MS	[27]
2002	2-Octanol	1.1	SPE	GC-ion trap MS	[28]
2004	2-Octanol	0.60	SPE	GC-ion trap MS	[15]
2005	2-Methyl-1-pentanol	0.98	Liquid–liquid microextraction or SPE	GC-single quadrupole MS	[29]
2006	4-Heptanolide	3	HS-SPME	GC-ion trap MS	[30]
2009	^2^H_7_-γ-Nonalactone	0.1	SPE	GC-single quadrupole MS	[3]
2013	^2^H_7_-γ-Decalactone	0.1	HS-SPME	GC-triple quadrupole MS	[18]
2014	γ-Heptalactone, 3,4-dimethylphenol	2.11	HS-SPME	GC-ion trap MS	[16]
2020	1-Menthol	0.1	SPE	GC-triple quadrupole MS	[5]
2023	^2^H_2_^13^C_2_-γ-Nonalactone	0.4	SPE	GC-single quadrupole MS	This work

Twelve NZ Pinot noir samples were subsequently analysed in triplicate using the above method and concentrations of γ-nonalactone **1** in each sample were calculated. The results of these analyses (means of triplicates and their standard) are shown in Table 2. The data collected for each wine concerning their vintage, region of production, and price are provided in Table S7. All wines had concentrations of **1** well above the LOD and LOQ for this analytical method. Interestingly, however, none of the samples exceeded the reported ODT of γ-nonalactone **1** (30 µg L^−1^). The highest concentration was measured in PN_4, with 22.5 µg L^−1^. The range of concentrations reported in the current work (8.30–22.5 µg L^−1^) is on par with concentrations measured in other studies of Pinot noir aroma; 10–18 µg L^−1^ γ-nonalactone **1** was reported in Oregon Pinot noir wines made from grapes of different maturities, from two consecutive vintages [31]; 6.9–14.8 µg L^−1^ in Pinot noir made with grapes subjected to different levels of dehydration [32]; and < 0.1–34.8 µg L^−1^ in Australian Pinot noir samples [3].

**Table 2:** Concentrations of γ-nonalactone measured in 12 commercial NZ Pinot noir wines

Sample name	γ-Nonalactone concentration (µg L^−1^)	Standard deviation ( ±)
PN_1	11.18	0.02
PN_2	8.74	0.05
PN_3	9.93	0.01
PN_4	22.49	0.01
PN_5	9.10	0.03
PN_6	12.49	0.01
PN_7	12.90	0.01
PN_8	8.30	0.02
PN_9	9.72	0.05
PN_10	12.59	0.02
PN_11	9.90	0.01
PN_12	8.76	0.03

Very high concentrations of γ-nonalactone exceeding 150 µg L^−1^ were reported in Oregon Pinot noir, five times the ODT of this compound. This study assessed the impacts of different levels of vine fruit zone leaf removal, in two consecutive vintages. γ-Nonalactone concentration was not associated with leaf removal [17]. The reason for these contrasting results is unclear, and merits further investigation, particularly considering that other studies looking at Oregon Pinot noir have reported similar concentrations to those reported in the current work [26, 27].

Although below the ODT, it has been reported that there are synergistic interactions between γ-nonalactone and other linear aliphatic lactones. Even when present below their respective ODTs, a strong synergism was shown to exist between γ-octalactone, γ-nonalactone, γ-decalactone, γ-undecalactone, and γ-dodecalactone, resulting in a significantly higher odour activity than the addition of their concentrations relative to respective ODTs would predict [10]. A similar phenomenon was discovered in reconstitution studies of Australian Viognier, where lactones including γ-nonalactone were shown to significantly increase the perception of apricot aroma, when monoterpenes were present. This suggests that some additional synergy exists between γ-nonalactone and other aroma compounds [33]. Clearly, the impact of aroma compounds in wine is more complex than simply assessing their ODT, and despite γ-nonalactone being present below its ODT in all Pinot noir samples analysed in this work, their impact on NZ Pinot noir aroma could still be significant.

**Fig. 3:**
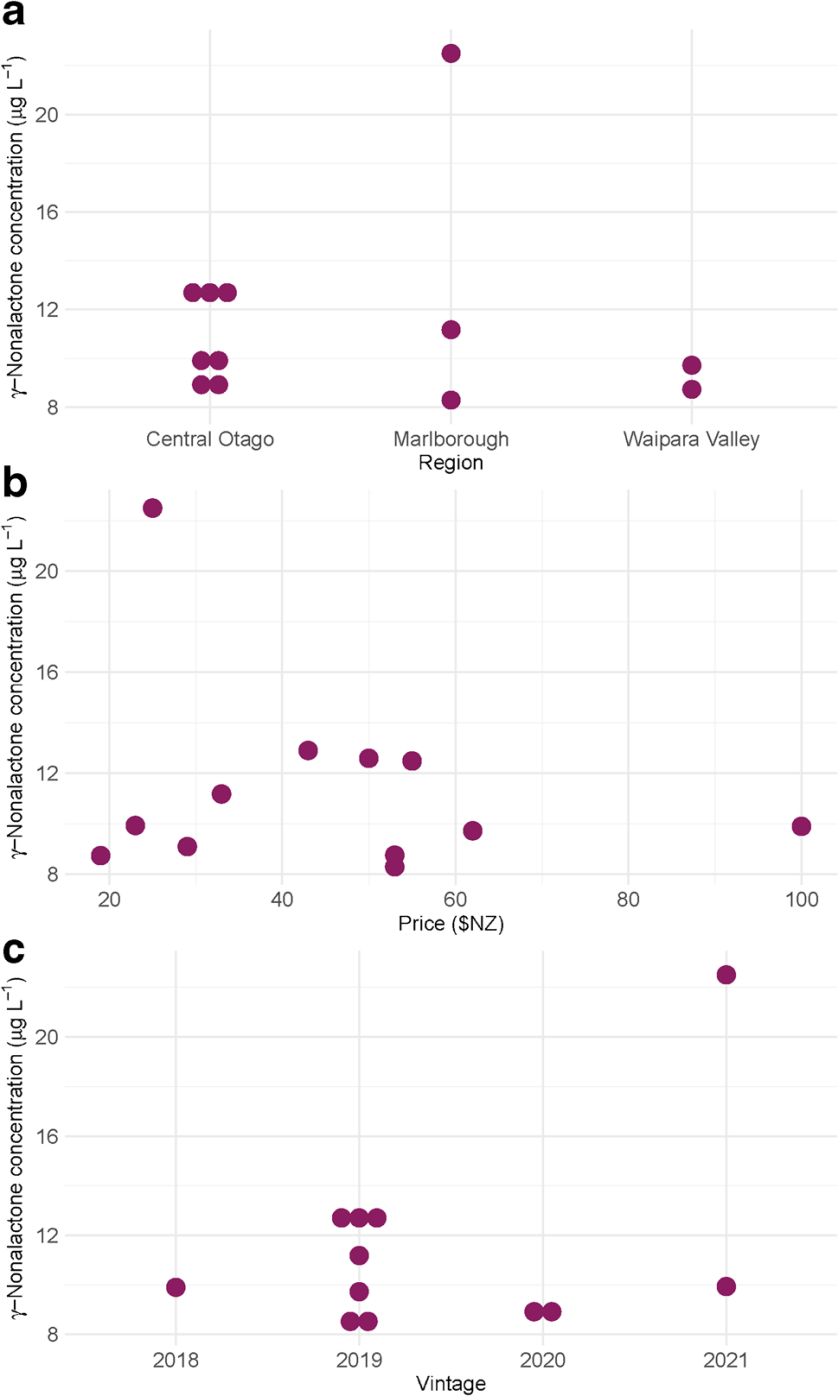
Concentrations of γ-nonalactone **1** (µg L^−1^) in NZ Pinot noir samples arranged by **a** region; **b** vintage; and **c** price ($NZ)

The effect of vintage, region of origin, and price on the concentration of γ-nonalactone **1** for these samples was explored. There was no observable trend for price and vintage, nor difference depending on region (Fig. 3a–c). These factors were formally assessed by multiple regression analysis, with sequential removal of non-significant variables (*p*-value > 0.05) to find any significant associations (*p*-value < 0.05). No significant associations were found between any of these variables and γ-nonalactone **1** concentration, suggesting that differences in γ-nonalactone **1** concentrations are largely due to other factors. Based on the knowledge of variables that influence γ-nonalactone **1**, such factors could include viticultural factors, the yeast strain used for fermentation, or the type/extent of oak influence utilised in winemaking [2, 29-31]. Additionally, as this set of data is relatively small, significant associations between price, region, vintage, and γ-nonalactone **1** concentration may be uncovered with the use of a larger dataset.

Investigation of any contribution of γ-nonalactone to undesirable prune aroma in NZ Pinot noir aroma also merits further investigation. This work did not involve any sensory analysis; however, it would be useful to determine if there were any links between higher concentrations of γ-nonalactone **1** and increased prune aroma, indicative of premature ageing of red wines [4]. This work also did not investigate Pinot noir wines older than 2018; it would be interesting to investigate whether older examples of NZ Pinot noir wines exhibited higher concentrations of γ-nonalactone **1**.

In conclusion, the synthesised novel ^2^H_2_^13^C_2_-γ-nonalactone **15** is a suitable internal standard for the accurate and precise quantification of γ-nonalactone **1** in red wines through the SIDA-SPE-GC–MS method reported herein, as it is stable under wine spiking and SPE conditions. Although similar LODs for γ-nonalactone **1** in wine have been reported in previous literature (Table 1), using an isotopically labelled internal standard as opposed to a surrogate internal standard is beneficial as it is anticipated that an isotopically labelled standard will behave similarly to the analyte of interest, even when used in a variety of matrices. The results of this study suggest that the concentration of γ-nonalactone **1** in NZ Pinot noir wines is not correlated with vintage and price or influenced by grape growing region, and instead other factor/s are likely to be involved in the modulation of γ-nonalactone **1** concentration. The concentrations of γ-nonalactone **1** in the samples in this study are in agreement with those found previously in Pinot noir wines, except for those found in one study looking at Oregon Pinot noir, which reported much higher concentrations. Investigation into the biogenesis of γ-nonalactone **1** during wine production may help to shed light on the origins of this compound, and the drivers of variability in concentration between different wines.

## Supplementary Information

Below is the link to the electronic supplementary material.

Supplementary file1 (PDF 493 kb)
