# Supplementary material for: Synthesis of a novel isotopically labelled standard for quantification of γ-nonalactone in New Zealand Pinot noir via SIDA-SPE-GC–MS
Source: Anal Bioanal Chem. 2023 Jun 13;415(20):5035–47. doi: 10.1007/s00216-023-04789-2 (PMC10386917; doi:10.1007/s00216-023-04789-2)
Supplement: Supplementary file 1 — Supplementary file1 (PDF 493 kb) [file 216_2023_4789_MOESM1_ESM.pdf]

# Synthesis of a Novel Isotopically Labelled Standard for Quantification of γ-Nonalactone in New Zealand Pinot noir via SIDA-SPE-GC-MS

Gillean Miller[1] (ORCID: 0000-0002-1806-8826), David Barker[1,2] (ORCID: 0000-0002-3425-6552), Lisa Pilkington[1,3] (ORCID: 0000-0002-9292-3261), Rebecca C. Deed[1,4,5] (ORCID: 0000-0001-6121-6786)

## Supplementary Information

## Wine Technical Data

**Table S1: Measured RS, TA, free and total $SO_2$ of wine samples in this work**

| Sample | Alcohol (% v/v) | RS (g $L^{-1}$) | TA (g $L^{-1}$) | pH | Free $SO_2$ (ppm) | Total $SO_2$ (ppm) |
|---|---|---|---|---|---|---|
| **PN_1** | 12.3 | 0.244 | 4.85 | 3.49 | 3.70 | 31.4 |
| **PN_2** | 12.7 | -0.146 | 4.72 | 3.60 | 5.78 | 16.1 |
| **PN_3** | 13.2 | 0.491 | 5.13 | 3.71 | 12.6 | 52.0 |
| **PN_4** | 13.0 | 0.679 | 5.35 | 3.67 | 11.3 | 45.5 |
| **PN_5** | 12.7 | -0.0894 | 4.94 | 3.72 | 7.90 | 22.8 |
| **PN_6** | 13.9 | 5.48 | 4.90 | 3.61 | 9.40 | 41.1 |
| **PN_7** | 12.7 | 0.514 | 4.94 | 3.64 | 9.20 | 29.6 |
| **PN_8** | 12.5 | 0.461 | 4.35 | 3.60 | 7.52 | 35.7 |
| **PN_9** | 14.1 | 1.78 | 5.42 | 3.71 | 8.61 | 30.9 |
| **PN_10** | 13.2 | 0.439 | 5.12 | 3.60 | 8.59 | 32.7 |
| **PN_11** | 13.5 | 2.48 | 5.03 | 3.61 | 8.81 | 56.1 |
| **PN_12** | 13.6 | 2.03 | 4.89 | 3.94 | 8.46 | 45.8 |
| **Dry red cask wine** | 11.9 | 5.48 | 6.13 | 3.46 | 24.2 | 106 |

## $^{2}H_6$-γ-Nonalactone Synthesis

*Potassium 4-hydroxynonanoate* **2***.* To a stirred solution of γ-nonalactone **1** (2.49 g, 15.43 mmol) in methanol (30 mL) was added potassium hydroxide (<85 % purity, 1.12 g, 15.43 mmol). This mixture was stirred at room temperature for 3 days. The solvent was removed *in vacuo* to yield the *title compound* **2** (3.22 g, 98 %) as a white solid which was used in the following reaction without further purification.

**$^{1}$H NMR (400 MHz, $^{2}H_2O$)** 0.89 (3H, t, *J* = 6.6 Hz, H9), 1.24-1.58 (8H, m, H-5, H-6, H-7, H-8), 1.62-1.84 (2H, m, H-3), 2.17-2.36 (2H, m, H-2), 3.61-3.71 (1H, m, H-4). $^{1}$H NMR was in agreement with literature values.[3]

*Isopropyl 4-hydroxynonanoate* **3***.* Potassium 4-hydroxynonanoate **2** (1.00 g, 4.71 mmol) was dissolved in DMSO (30 mL) with heating. The resulting solution was cooled to room temperature, then isopropyl bromide (3.70 mL, 23.00 mmol) was added. This mixture was stirred at room temperature for 2 days, then diluted with water (20 mL) and extracted with diethyl ether (3 x 15 mL portions), dried with anhydrous magnesium sulfate and concentrated *in vacuo*. The resulting oil was purified by column chromatography (5 % v/v $Et_2O/CH_2Cl_2$) to give the *title compound* **3** (0.484 g, 47 %) as a pale-yellow oil.

**$^{1}$H NMR (400 MHz, $C^{2}HCl_3$)** 0.87 (3H, t, *J* = 6.8, H-9), 1.21 (6H, d, *J* = 6.3 Hz, H-2'), 1.22-1.47 (8H, m, H-5, H-6, H-7 and H-8), 1.60-1.85 (2H, m, H-2), 2.33-2.46 (2H, m, H-3), 3.42-3.74 (1H, m, H-4), 5.00 (1H, sept, *J* = 6.3 Hz, H-1'). $^{1}$H NMR was in agreement with literature values.[3]

*Isopropyl 4-oxononanoate* **4***.* Oxalyl chloride (0.25 mL, 2.88 mmol) was added to a mixture of DMSO (0.41 mL, 5.7 mmol) and $CH_2Cl_2$ (9 mL) at – 78 °C under an atmosphere of nitrogen, over 2 mins. This mixture was stirred at – 78 °C for 30 mins, then a solution of isopropyl 4-hydroxynonanoate **3** (0.40 g, 1.85 mmol) in $CH_2Cl_2$ (1.5 mL) was added and the resulting mixture was stirred at – 78 °C for 45 mins. Triethylamine (1.6 mL, 11.6 mmol) was added slowly, and this mixture was stirred at – 78 °C for 30 mins, then 0 °C for 30 mins, then room temperature for 30 mins. The reaction mixture was poured into a rapidly stirred solution of $NaHSO_4$ (1.0 M, 12 mL), the layers separated, and the aqueous layer was extracted with $CH_2Cl_2$ (3 x 10 mL portions). The combined organic extracts were concentrated under reduced pressure, and the resulting residue was taken up in diethyl ether (10 mL) and washed with a 1.0 M solution of $NaHSO_4$ (3 x 5 mL portions), water (5 mL), saturated $NaHCO_3$ (5 mL) and brine (5 mL). The organic phase was dried with anhydrous magnesium sulfate, filtered, and concentrated *in vacuo* to

yield the crude product as a pale-yellow oil. This oil was purified by column chromatography (1:1 ethyl acetate: petroleum ether) to give the *title compound* **4** (0.328 g, 83 %) as a pale-yellow oil.

**$^1$H NMR (400 MHz, C$^2$HCl$_3$)** 0.88 (3H, t, *J* = 7.0 Hz, H-9), 1.22 (6H, d, *J* = 6.3 Hz, H-2'), 1.25-1.32 (4 H, m, H-7, H-8), 1.58 (2H, qn, *J* = 7.2 Hz, H-6), 2.43 (2H, t, *J* = 7.5 Hz, H-5), 2.53 (2H, t, *J* = 6.5 Hz, H-2), 2.71 (2H, t, *J* = 6.7 Hz, H-3), 4.98 (1H, sept, *J* = 7.4 Hz, H-1'). $^1$H NMR was in agreement with literature values.[3]

*$^2H_6$-Oxononanoic acid* **5**. Isopropyl 4-oxononanoate **4** was heated under reflux in 20 % (w/v) $^2$HCl/$^2H_2O$ solution (2.3 mL) and $^2H_2O$ (4 mL) under nitrogen for 7 days. This reaction mixture was allowed to cool and was then extracted with diethyl ether (3 x 6 mL portions). The organic extracts were combined, then dried with anhydrous magnesium sulfate, filtered, and concentrated *in vacuo*. The resulting residue was again heated under reflux in 20 % (w/v) $^2$HCl/$^2H_2O$ solution (2.3 mL) and $^2H_2O$ (4 mL) under nitrogen for 7 days. This reaction mixture was allowed to cool and was then extracted with diethyl ether (3 x 6 mL portions). The organic extracts were combined, then dried with anhydrous magnesium sulfate, filtered, and concentrated *in vacuo* to give the *title compound* **5** (0.234 g, 93 %) as a yellow oil.

**$^1$H NMR (400 MHz, C$^2$HCl$_3$)** 0.86 (3H, t, *J* = 7.1 Hz, H-9), 1.23-1.32 (4H, m, H-7, H-8), 1.55 (2H, *t*, J = 7.0 Hz, H-6), 2.35-2.44 (0.29H*, m, H-5), 2.46-2.60 (1.83H*, m, H-2), 2.62-2.71 (0.30H*, m, H-3). $^1$H NMR integrals denoted * represent partially deuterated positions.

**$^{13}$C NMR (100 MHz, C$^2$HCl$_3$)** 13.9 (CH3, C-9), 22.4 (CH2, C-8), 23.4 (CH2, C-6), 27.5 (CH2, C-2), 31.3 (CH2, C-7), 36.6 (CH2, C-3), 58.2 (CH2, C-3), 175.9 (COOH, C-1)

***m/z* (ESI$^+$)** 173 (MH+, 100 %), 174 (10), 175 (2), 131 (1)

**HRMS** Found (MH$^+$): 173.1185, $C_9H_{17}O_3^+$ requires 173.1178ᵗ

ᵗThe most abundant molecular ion of a mixture of analogues.

*$^2H_6$-γ-Nonalactone* **6**. $^2H_6$-oxononanoic acid **5** (0.230 g, 1.28 mmol) was added to a solution of sodium borohydride (0.129 g, mmol) in $^2H_2O$ (6 mL), and this mixture was stirred under nitrogen at room temperature for 24 h. This reaction was quenched by the careful addition of 20 % (w/v) $^2$HCl/$^2H_2O$ solution (pH 2), then stirred under nitrogen at room temperature for an additional 24 h. The mixture was extracted with diethyl ether (3 x 15 mL portions), then the combined organic extracts were dried with anhydrous magnesium sulfate and

concentrated *in vacuo* to give the crude product. The crude product was purified by column chromatography (1:1 diethyl ether: petroleum ether) to give the *title compound* **6** (73.5 mg, 35 %) as a colourless oil.

**Rf (1:1 diethyl ether: petroleum ether)** 0.36

**IR: $\upsilon_{max}$(film)/cm$^{-1}$** 2927 and 2861, 1767, 1634, 1522, 1467 and 1424, 1336, 1264 and 1193, 1132, 1024 and 902

**$^1$H NMR (400 MHz, $C^2HCl_3$)** 0.89 (3H, t, *J* = 6.7 Hz, H-9), 1.25- 1.50 (6H, m, H-6, H-7 and H-8), 1.52-1.77 (0.99H*, m, H-5) 1.78-1.88 (0.18H*, m, H-3a) 2.24-2.34 (0.20H*, m, H-3b), 2.47-2.56 (1.74H*, m, H-2), 4.46 (1H, s, H-4).

$^1$H NMR integrals denoted * represent partially deuterated positions.

**$^{13}$C NMR (100 MHz, $C^2HCl_3$)** 14.0 (CH3, C-9), 22.5 (CH2, C-6, C-7, or C-8), 24.8 (CH2, C-6, C-7, or C-8), 28.8 (CH2, C-2), 31.5 (CH2, C-6, C-7 or C-8), 80.9 (CH, C-4), 177.3 (COO, C-1)

***m/z* (ESI$^+$)** 239 (100 %), 223 (20), 181 (2), 157 (MH+, 3), 144 (5), 133 (3)

**HRMS** Found (MH$^+$): 157.1159, $C_9H_{17}O_2^+$ requires 157.1229$^\dagger$

$^\dagger$The most abundant molecular ion of a mixture of analogues.

## Retention Times

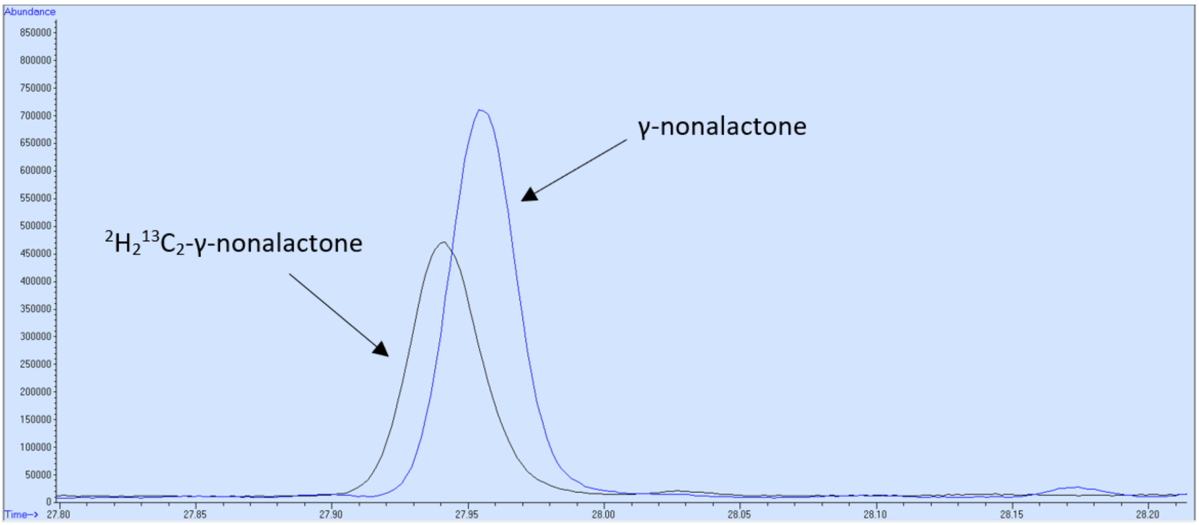


**Fig. S1: Excerpt of chromatogram showing base peaks corresponding to γ-nonalactone 1 and $^{2}H_{2}{}^{13}C_{2}$-γ-nonalactone 1**

## Ions used For Quantification

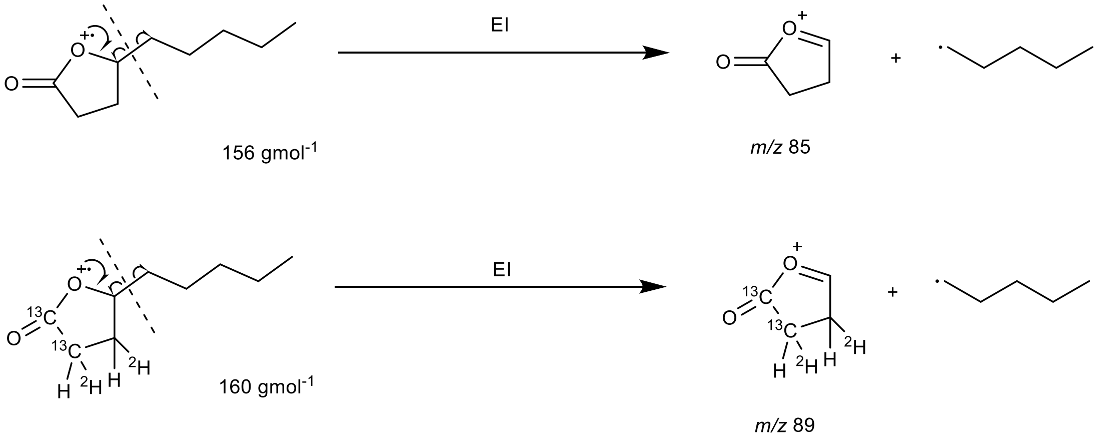


**Scheme S1: Hypothesised fragmentation pathway of (a) γ-nonalactone and (b) $^{2}H_{2}{}^{13}C_{2}$-γ-nonalactone to produce their respective base peaks during EI mass spectrometry**

## Characterisation of $^2H_6$-Oxononanoic acid 5

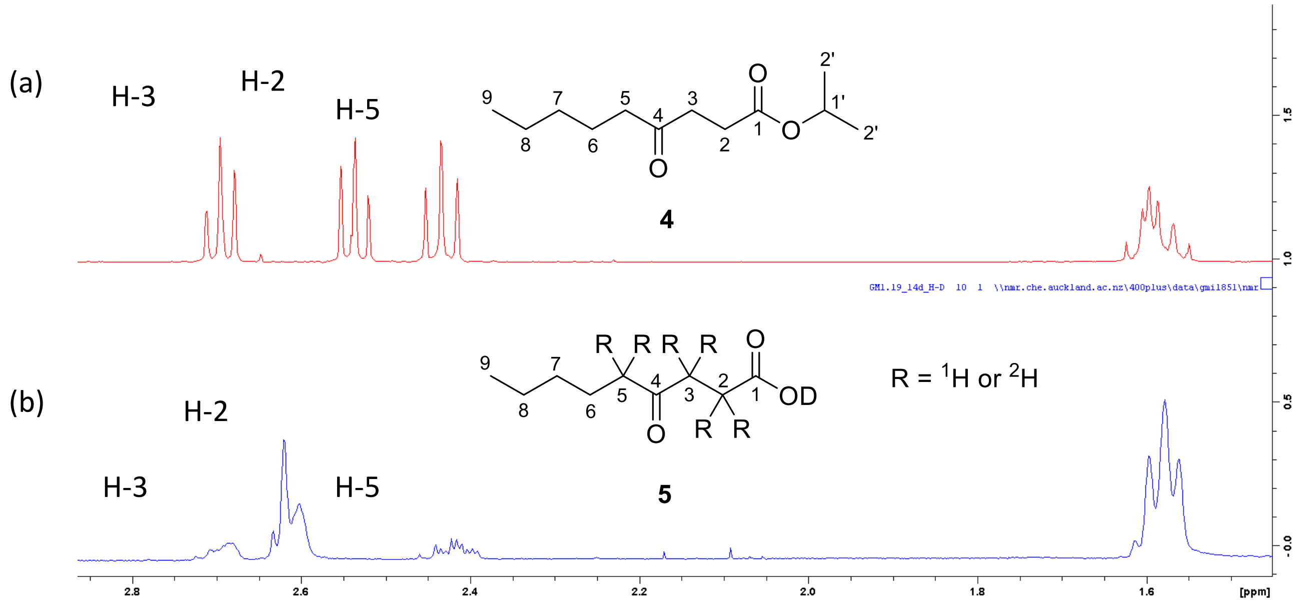


**Fig. S2: Excerpts from $^1$H NMR spectra of (a) isopropyl 4-oxononanoate 4 prior to deuterium exchange and (b) 4-oxononanoic acid 5 after 14 days deuterium exchange reaction conditions. Peaks corresponding to exchangeable protons are shown (H-2, H-3, and H-5). Incomplete deuterium exchange is shown by remaining peaks corresponding to these protons**

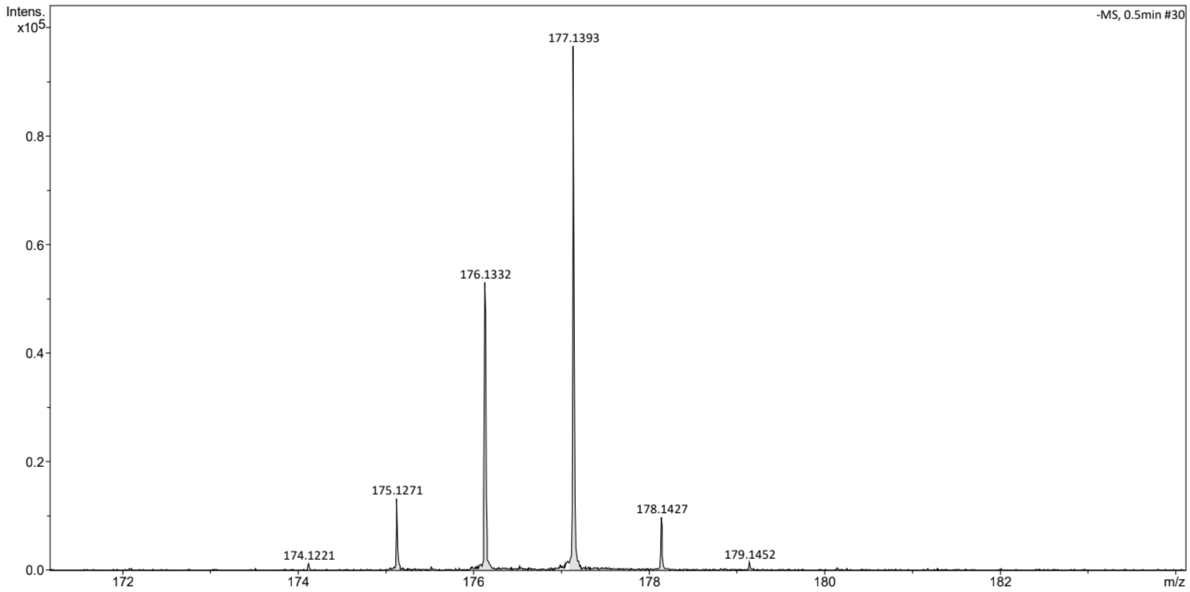


**Fig. S3: Excerpt from HRMS analysis (negative ion mode) of 4-oxononanoic acid 5 after 14 days of deuterium exchange conditions. *m/z* 177 corresponds to full deuterium exchange ($C_9H_9{}^2H_6O_3^-$), whilst the presence of *m/z* 176 and *m/z* 175 likely indicate incomplete exchange ($C_9H_{10}{}^2H_5O_3^-$ and $C_9H_{11}{}^2H_4O_3^-$, respectively)**

## Verification of suitable SIDA internal standard

**Table S2: Analysis of isotopologue distribution of $^{2}H_{2}{}^{13}C_{2}$-γ-nonalactone after different model wine spiking conditions. "M" refers to a *m/z* ratio of 85, corresponding to the natural analogue of γ-nonalactone. The relative integrals of M+2, M+3 and M+4 (corresponding to *m/z* 87, 88 and 89, respectively) were monitored and are shown here**

| | **Peak areas corresponding to specified *m/z* ratio** | | | | | **Relative peak area of specified *m/z* ratios** | | |
|---|---|---|---|---|---|---|---|---|
| **Sample** | **M+0** | **M+1** | **M+2** | **M+3** | **M+4** | | | |
| | **85** | **86** | **87** | **88** | **89** | **M+4/M+3** | **M+4/M+2** | **M+3/M+2** |
| **30min_RT_a** | 1417 | 1221 | 8053 | 27753 | 35838 | 1.29 | 4.45 | 3.45 |
| **30min_RT_b** | 809 | 867 | 7882 | 27653 | 35237 | 1.27 | 4.47 | 3.51 |
| **30min_RT_c** | 689 | 932 | 8950 | 30254 | 39822 | 1.32 | 4.45 | 3.38 |
| **30min_HEAT_a** | 481 | 915 | 7301 | 25221 | 32520 | 1.29 | 4.45 | 3.45 |
| **30min_HEAT_b** | 564 | 767 | 6037 | 20009 | 26790 | 1.34 | 4.44 | 3.31 |
| **30min_HEAT_c** | 492 | 1151 | 7285 | 26139 | 35354 | 1.35 | 4.85 | 3.59 |
| **180min_RT_a** | 721 | 898 | 7371 | 26458 | 34704 | 1.31 | 4.71 | 3.59 |
| **180min_RT_b** | 568 | 1197 | 9198 | 31786 | 41949 | 1.32 | 4.56 | 3.46 |
| **180min_RT_c** | 196 | 1614 | 8981 | 32240 | 43309 | 1.34 | 4.82 | 3.59 |
| **180min_HEAT_a** | 533 | 813 | 6023 | 21328 | 27790 | 1.30 | 4.61 | 3.54 |
| **180min_HEAT_b** | 301 | 1017 | 6720 | 22613 | 28806 | 1.27 | 4.29 | 3.367 |
| **180min_HEAT_c** | 521 | 541 | 4941 | 17694 | 22741 | 1.29 | 4.60 | 3.58 |

## Calibration Comparison and Verification

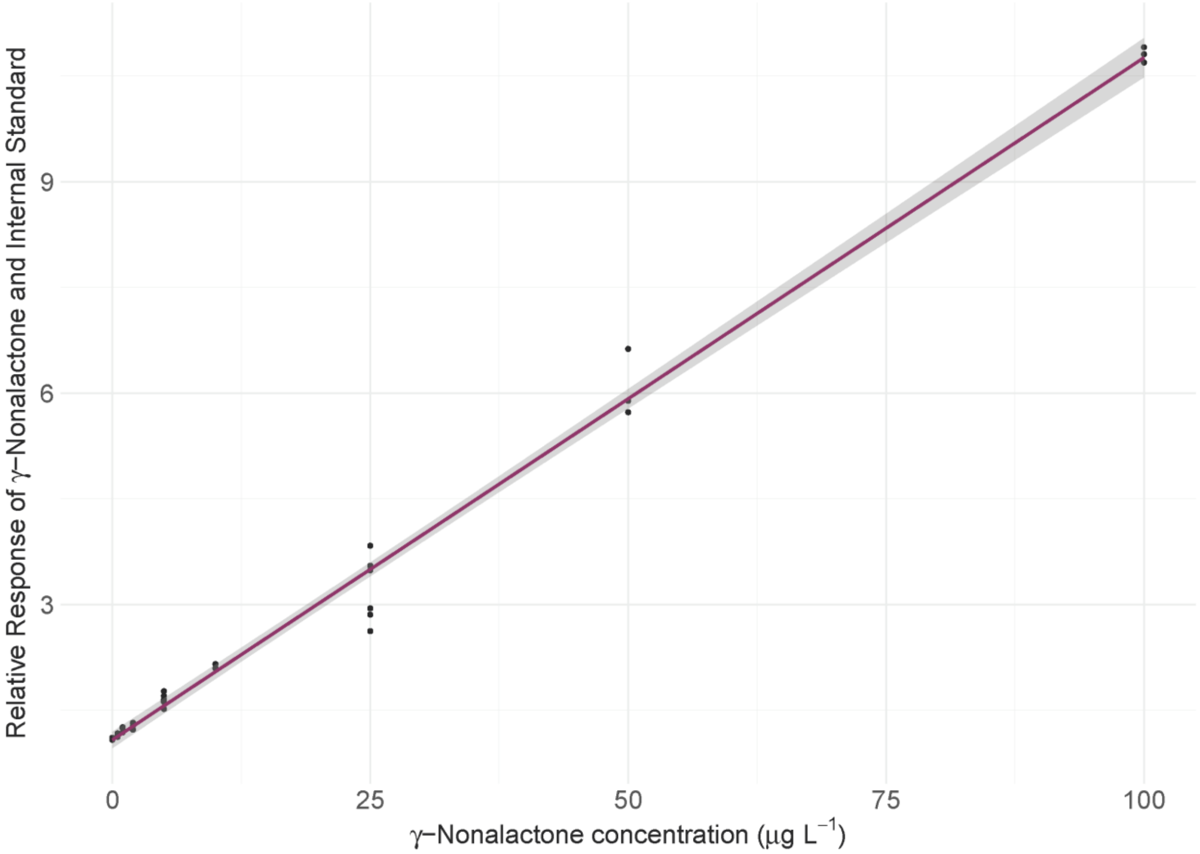


**Fig. S4: Calibration curve constructed using a dry red wine matrix, showing relative response of γ-nonalactone 1 and $^{2}H_{2}{}^{13}C_{2}$-γ-nonalactone 15 with varying concentrations of γ-nonalactone**

**Table S3: Dry red wine calibration data**

| | Dry Red Wine Calibration Data |
|---|---|
| **slope** | 0.09684 |
| **slope standard error** | 0.00159 |
| **degrees of freedom** | 32 |
| **95 % t-statistic for 99 df** | 2.037 |
| **95 % confidence interval (+/-)** | 0.00323883 |
| **Upper** | 0.10007883 |
| **lower** | 0.09360117 |
| **$R^2$** | 0.9914 |

**Table S4: Model Wine calibration data, with associated LOD and LOQ**

| | Model Wine Calibration Data |
|---|---|
| **slope** | 0.0958 |
| **slope standard error** | 0.000374 |
| **degrees of freedom** | 99 |
| **95 % t-statistic for 99 df** | 1.984 |
| **95 % confidence interval (+/-)** | 0.000742 |
| **upper** | 0.0966 |
| **lower** | 0.0951 |
| **$R^2$** | 0.999 |
| **a = estimate of intercept** | 0.0624 |
| **s = standard error of line** | 0.104 |
| **LOD = a+3.29s** | 0.406 |
| **LOQ = a+10s** | 1.11 |

**Table S5: Relative response of γ-nonalactone and $^{2}H_2{}^{13}C_2$-γ-nonalactone in instrumental repeats. This data was used to find the repeatability of this analytical method**

| Sample | Relative Response of γ-nonalactone and $^{2}H_2{}^{13}C_2$-γ-nonalactone |
|---|---|
| **MWI_5a_1** | 0.543 |
| **MWI_5a_2** | 0.515 |
| **MWI_5a_3** | 0.527 |
| **MWI_5a_4** | 0.523 |
| **MWI_5a_5** | 0.539 |
| **MWI_5a_6** | 0.537 |
| **MWI_5a_7** | 0.535 |
| | |
| **n** | 7 |
| **Standard deviation** | 0.0101 |
| **Reproducibility** | 0.00380 |
| **% Reproducibility** | 0.380 |

**Table S6: Relative response of γ-nonalactone and $^{2}H_{2}{}^{13}C_{2}$-γ-nonalactone in experimental repeats. Repeats were conducted on different days (indicated by letters a, b, c, d, and e) and in triplicate (indicated by numbers 1, 2 and 3). * Represents outliers, detected by Grubbs' test and these were excluded from averages**

| **Sample** | **Relative Response of γ-nonalactone and $^{2}H_{2}{}^{13}C_{2}$-γ-nonalactone** | Average |
|---|---|---|
| **MWI_5r_1a** | 0.577 | 0.577 |
| **MWI_5r_2a** | 0.586 | |
| **MWI_5r_3a** | 0.568 | |
| **MWI_5r_1b** | 0.619 | 0.588 |
| **MWI_5r_2b** | 0.554 | |
| **MWI_5r_3b** | 0.591 | |
| **MWI_5r_1c** | 0.0104* | 0.569 |
| **MWI_5r_2c** | 0.576 | |
| **MWI_5r_3c** | 0.562 | |
| **MWI_5r_1d** | 0.545 | 0.537 |
| **MWI_5r_2d** | 0.523 | |
| **MWI_5r_3d** | 0.538 | |
| **MWI_5r_1e** | 0.547 | 0.542 |
| **MWI_5r_2e** | 0.516 | |
| **MWI_5r_3e** | 0.564 | |
| | | |
| **n** | 14 | |
| **Standard deviation** | 0.0269 | |
| **Repeatability** | 0.00720 | |
| **% Repeatability** | 0.720 | |

## Wine Data

**Table S7: Relative response of γ-nonalactone and $^{2}H_2{}^{13}C_2$-γ-nonalactone for each NZ Pinot noir sample, measured in triplicate and averaged. The calculated γ-nonalactone concentration, vintage, region, and price ($NZ) are also provided for each wine. * Represents outliers, detected by Grubbs' test and these were excluded from averages**

| | **Relative response γ-Nonalactone/Internal Standard Area** | | | | | | | | |
|---|---|---|---|---|---|---|---|---|---|
| **Sample** | **Replicate 1** | **Replicate 2** | **Replicate 3** | **Average** | **Calculated γ-Nonalactone Concentration (μg $L^{-1}$)** | **Standard deviation (±)** | **Vintage** | **Region** | **Price ($NZ)** |
| **PN_1** | 1.13 | 1.15 | 1.12 | 1.13 | 11.2 | 0.0183 | 2019 | Marlborough | 32.99 |
| **PN_2** | 0.864 | 0.875 | 0.961 | 0.900 | 8.74 | 0.0532 | 2020 | Waipara Valley | 18.99 |
| **PN_3** | 1.06 | 2.31* | 1.06 | 1.01 | 9.93 | 0.00359 | 2021 | Central Otago | 22.99 |
| **PN_4** | 2.23 | 2.22 | 2.21 | 2.22 | 22.5 | 0.0100 | 2021 | Marlborough | 24.99 |
| **PN_5** | 0.964 | 0.923 | 0.915 | 0.934 | 9.10 | 0.0265 | 2020 | Central Otago | 28.99 |
| **PN_6** | 1.27 | 1.26 | 1.24 | 1.26 | 12.5 | 0.0132 | 2019 | Central Otago | 54.99 |
| **PN_7** | 1.31 | 1.29 | 1.30 | 1.30 | 12.9 | 0.00728 | 2019 | Central Otago | 42.99 |
| **PN_8** | 0.848 | 0.847 | 0.878 | 0.858 | 8.30 | 0.0178 | 2019 | Marlborough | 52.99 |
| **PN_9** | 0.957 | 1.03 | 0.497* | 0.994 | 9.72 | 0.0530 | 2019 | Waipara Valley | 61.99 |
| **PN_10** | 1.25 | 1.27 | 1.29 | 1.27 | 12.6 | 0.0208 | 2019 | Central Otago | 49.99 |
| **PN_11** | 1.01 | 1.02 | 1.01 | 1.01 | 9.90 | 0.00622 | 2018 | Central Otago | 99.99 |
| **PN_12** | 0.932 | 0.870 | 0.902 | 0.902 | 8.76 | 0.0309 | 2019 | Central Otago | 52.99 |
